# Essays on the Determination of Blood to the Head

**Published:** 1844-01

**Authors:** 


### Art. XIII.

*Essays on Determination of Blood to the Head.* By Robert Hull, m.d. Physician to the Norfolk and Norwich Hospital.—*Norwich,* 1842. 8vo, pp. 200.

We had supposed, after the investigations of Dr. Abercrombie and other labourers in the same field of pathological inquiry, that scarcely any professional man could be so ignorant as not to know that the symptoms, vulgarly supposed to be indicative of an overflow of blood to the head, that is to say, of increased vascularity of the brain, are common to very opposite states of that organ. But in fact, if we go back to the ancient authorities in medicine, we shall find in their works undoubted evidence that they were perfectly aware of this fact, and that they applied remedies in such cases upon this principle with considerable tact and talent for discrimination. Thus, Galen divides headaches into acute and chronic, and subdivides each into several varieties; as for example, cephalalgia from a hot and from a cold intemperament; cephalalgia from plethora, to be relieved by venesection; and the same from sympathy with the stomach and liver, to be cured by emetics and cholagogues. Haly Abbas makes mention of intense headach from sympathy with the uterus, after miscarriages, &c. The ancient authorities also treat of the lethargic affections in such a manner as shows that they did not set them all down as being the same in kind and requiring no diversity of treatment. But still, notwithstanding all the information to be derived from professional authorities, both ancient and modern, it must be admitted that there is ample room for further improvement in the pathology and therapeutics of this class of diseases, as cases but too frequently occur in practice which puzzle the most sagacious and intelligent practitioner to determine the nature of the disease, and point out the line of treatment most suitable to it. Whether the work under review contributes anything to improve our knowledge on these important points, our readers will be best able to judge after we have laid before them a short analysis of its contents.

After a dedication to Sir Henry Halford in the author's favorite style, of which we shall say more hereafter, he proceeds in his *Essay introductory* to unfold his views with regard to the preciousness of blood in the animal economy, and to expatiate in strong terms on the sin and shame of shedding it in a reckless Sangradic manner.

There is too much truth in the following graphic picture of our author; but we have reason to complain that here, as elsewhere, he fails to point out any rules for distinguishing those states which, although resembling each other, demand very different modes of treatment.

"I wish to impress upon the student, that in medical, as well as in judicial conditions, the doubt should tell in favour of the pannel. So sacred is blood, do not spill it upon conjecture. What is the history of our art in the matter of traumatic apoplexies produced by fractured crania or depressed bone? Death! death! But never without previous bleedings! A poor fellow is brought into an hospital stupid, haggard, *demanding commiseration* through his mere wretchedness of look. He has received a blow on his skull. 'His brain is concussed.' Yes. But every other part is concussed. 'Do, pray, let him rest! Do not bleed him! Do not leech him!' But repose is not the order of the day; and, as something must be *done,* the patient is teazed with leeches, blisters, mercury: chatterboxes inquiring how he feels; nurses asking him what he wants! Then comes reaction; attempts at victory by the vis medicatrix! But reaction is dreaded as much as the blow; and forthwith the lancet is employed to subdue *all* excitement, whether bodily or mental, whether heat of skin or garrulity of tongue. And it is used again and again until the patient expires. Then comes the post-mortem inspection; when it is found that the skull, somewhere or other, is split; that the membranes are yellow with lymph; serum effused; yet not over-much blood in the vessels. Then comes the consolation, that 'with such a state of parts no recovery was possible.' How do I know this? All I know is, that without blood no reparation of mischief can take place, that with free bloodletting meningitis *has* occurred. Surely such a sufferer could not have been worse if he had been let alone. Meddled with *thus*, can he have so fair a chance? Doubtless the brain is a most delicate organ; and lesions and inflammations must be combated with unusual activity. But the brain, like other structures, is under the regime of the vis medicatrix. It struggles long against mischief if you leave it power." (Introduction, p. xxv.)

We must not be carried too far, however, by the learned doctor's eloquent appeal. That in the first stage of injuries of the head, when the sensorium is oppressed and the vital energies sunk, it is highly improper to drain the vessels of blood we readily admit, and our best authorities in surgery are quite decided upon this point. But we never can admit it as a general rule of practice, that, *after reaction has taken place*, venesection ought not to be had recourse to, or that a patient so circumstanced has a better chance of recovery without loss of blood.

At pages 34, 35, and 36, he relates two cases of nervous irritability of the eyes, which, as they are happily illustrative of the pernicious effects of meddlesome surgery, and more especially of great depletion, we recommend to the attention of our younger readers.

*The first Essay* is on Vertigo, of which the author distinguishes several varieties: e.g. *Vertigo optica* of Darwin, to which persons about fifty or sixty years of age are subject, when their sight begins to fail; *vertigo symptomatica*, arising from sympathy with the stomach in dyspepsia and nervous diseases; *vertigo nervosa*, an affection peculiar to hysterical women, and men who over-work the brain; *vertigo ebriorum*, experienced by drunkards, in whose case Dr. Hull maintains with Dr. Percy, that the influence of the drink is exerted directly on the brain; the *vertigo of sea-sickness*, best prevented, he says, by the horizontal posture and closed eyelids, and for which he recommends "strong spirit and water, and if this avail not, a powerful opiate." Few will question the correctness of the conclusion to which Dr. Hull arrives, "that vertigo is not always indicative of a determination to the brain;" and if he had turned his knowledge of Greek to cultivating an acquaintance with Galen and Aretæus, as well as with Euripides and Pindar, he would have learned that it is not true that "giddiness is almost always attributed to a fulness of the vessels of the brain." On the contrary, even these ancient authors were aware that it often arises from sympathy with distant parts, as the stomach, the liver, and the spleen; though notwithstanding what Dr. Hull has written *in favour* of vertigo, we must say that in too many cases we have found reason to agree with Aretæus, in looking upon it as a *serious complaint*.

*The second Essay* On Sleepiness, contains some speculations on the *brain* and the *soul*, of so abstruse a nature, that we are almost afraid to meddle with them. What Dr. Hull says respecting the brain may be quite true, but for ordinary capacities, we think he jumps rather abruptly to the conclusion "that we think and digest with the same organ; solve a problem of Euclid; and dissolve a mutton-chop." As in the former essay, we desiderate in this the practical rules for discriminating cases requiring opposite kinds of treatment. At pp. 25-6, he relates two cases of apoplectic sleepiness, in the treatment of which, with all due deference to so high an authority, we are much disposed to think that he was wrong in the former, and "the other surgeon" right in the latter. Instead of quoting these cases, however, we will here present our readers, from our own observation, with a case which we consider important as showing that the most alarming symptoms of *apoplectic stupor*, may be altogether nervous.

A man about sixty years old, of a nervous temperament and timid disposition, while lying in bed half-a-sleep, was roused by a scream proceeding from an adjoining apartment; he started up and spoke a few words incoherently, but almost immediately became insensible, and had to be laid down in bed, when he soon fell into a state of the most profound stupor, insomuch that when he was bled in the arm by a surgeon about twelve hours after the attack, he showed not the slightest symptoms of sensibility. In this state he lay for the space of nearly three days, when he began to amend, and in the course of a few days his health became completely restored.

*The third Essay* is on SOPOR HYSTERICUS. This must be admitted to be a very interesting subject of inquiry, but we do not find many new facts brought forward by our author in relation to it. We also think that his plan of treatment will scarcely be admissible in those circles of society to which his friend Sir Henry Halford devotes his services. For example, (p. 28,) to rouse a girl from a profound hysterical sleep, he directed "to heat the kitchen poker to candescence, and brought the heated iron to approximation with her soles; near enough for the radiation; not near enough to burn. In a few seconds she felt the pain; retracted her feet; roused herself suddenly on her breech; rubbed her eyes; looked surprised around the room, and was well!" Fortunately, however, it is not necessary in all cases even to approximate the heated iron to the skin, as it often happens that when the patient hears this Boerhaavean order given, the terror reduces her to quiet.

*The fourth, fifth, sixth, and seventh Essays* treat of HEADACH, of which Dr. Hull describes several varieties, but without leaving us with the impression that he has exhausted the subject. Of course he is quite warranted in drawing the conclusion that pain in the head does not necessarily imply fulness of it; but this, as we have already said, is no new opinion, but has been maintained by the best authorities in medicine from the earliest ages. At the same time it must be admitted, that although, as Dr. Hull says, "the history of apoplexy is not a tale of headach," headach is, unquestionably, a very common precursor of it, and a not unfrequent attendant on its invasion. In plethoric headach it is admitted that "cupping is a commendable remedy." We believe it is so, generally speaking; we have, however, seen more than one instance where the subjects so treated have complained of feeling that more blood had been *drawn to* the head, than *taken from it* by the operation. In inflammatory and congestive affections of the head, we are, on the whole, disposed to prefer a revulsive bleeding from the arm. We have also seen much benefit from the application of a few leeches to the lining membrane of the nostrils. We believe that this mode of applying leeches is common in Italy, being in imitation of the old practice mentioned by Aretæus and Paulus Ægineta, that of scarifying the vessels of the nostrils. It has this additional advantage, that one leech thus applied will procure a greater discharge of blood than half a dozen applied to the external skin.

*The eighth Essay* is on Epileptic Convulsions, and the *ninth* on Apoplectic Convulsions. Though the former of these does not contain anything very original, we would beg to direct attention to the cases connected with *exostosis* of the cranium, and cured by the application of the trephine. That in epilepsy, bloodletting is generally very inefficacious, we do not require any new authority to convince us. In apoplectic convulsions the author admits that there is congestion in the brain; but suggests that "the cerebral infarction is venous;" and he maintains that even when it is well ascertained that there is congestion, it is not safe to apply the remedies usually relied upon, namely, purging and bleeding; as they have often the diametrically opposite effect from what they are intended to produce. From two cases which he relates, he draws the conclusion that "the purgative treatment, like bloodletting, may, when excessive, determine to the head," (p. 87;) and he says afterwards of bloodletting, "This case illustrates ....... the principle laid down by Dr. Warren, that the more you bleed the more you determine," (p 93.) If these conclusions are valid, we shall be obliged to forswear the lancet altogether, that we may not have the blood of our fellow-creatures upon our heads! In the preceding essays, Dr. Hull has proved to demonstration, that if we abstract blood when there is *no* "determination to the head," we shall most assuredly kill our patient, and now he tells us that we shall do so with still greater certainty if there be "determination," since "the more you bleed the more you determine."

*The tenth Essay* is on Delirium and Photopsia. Delirium is a symptom of so many diseases and different conditions of the system, that we do not conceive Dr. Hull warranted in affirming that "Delirium is a symptom for which bloodletting is very perniciously and at random prescribed, or rather practised." (p. 94.) On the contrary, we believe that not only do professional men not rashly bleed in cases attended with delirium unless there be other symptoms to indicate it, but that even the common people look upon delirium as being often connected with "emptiness of the brain." Dr. Hull's remarks in this chapter we, therefore, look upon as being uncalled for. His cases of *Photopsia* possess little interest.

In the *eleventh Essay*, on Insanity, he sets out with questioning the soundness of the established opinion, "that the disorder is more bodily than metaphysic (*mental?*)" an opinion, however, which we see nothing in his facts or arguments calculated to controvert. As Dr. Hull does not pretend to affirm that the profession is too bloodthirsty in the treatment of lunatics, so in this essay he has almost entirely lost sight of "determination to the brain." The cases he has collected on this head do indeed possess a certain degree of interest, as indeed every collection of carefully related cases cannot fail to do; but they do not at all bear on the subject of this work.

*The twelfth* and last *Essay* is on a characteristic subject, Death ; as if the author meant thereby to inculcate that this is the termination of "determination to the brain." His aim, however, in this essay is to establish the fact that the brain is the *ultimum moriens*, that it is well supplied with the vital fluid while all the other parts of the system are empty. Here he passes in review the dying scenes of some of the greatest worthies of ancient and modern times, Pericles, Hadrian, Epaminondas, Wolfe in Canada, Moore at Corunna, Nelson, "the Imperial Corsican," and last, (as if by way of contrast, we suppose,) a girl aged ten years, who dying of peritonitis, "occupied every interval, when not asleep, in singular and shrewd loquacity!!" Now all this may be very interesting to the collector of anecdotes ; but we do not see that these stories throw much light on the nature and treatment of "determination to the brain."

Before concluding, we must say a few words of the *manner* or style of Dr. Hull's book. Here it will be readily acknowledged there is no want of originality. We do not remember to have ever looked into a medical volume which contained so many words and expressions which were altogether new to us, or at all events used in a strange signification. Take the following as examples : *pigmeian* (pygmæan ?) p. x ; *libellule*, ib. ; *sanguifiction* (sanguifaction ?) p. xiv ; *practicality*, p. xvi ; *scirrhopöetic*, p. xxviii ; *laxation*, p. xxxvi ; *saginate*, p. 5 ; *metamorphiotic*, p. 27 ; *detegible*, p. 37 ; *arthric* (arthritic ?) p. 50 ; *salvatory*, p. 54 ; *diagnize*, p. 62 ; *hymenæous*, p. 68 ; *erotopoetic*, p. 69 ; *prepuberous*, p. 79 ; *feelable*, p. 90 ; *bibulous company*, p. 93 ; *empiricoid*, p. xliii ; *hysteroid*, p. 120 ; *despotules*, p. 128 ; *strategic*, p. 152, &c. &c. At first sight nothing struck us so remarkably in the appearance of the work as the profusion of Greek and Latin verses which we saw scattered over its pages. We had the curiosity to count the number of quotations from Euripides alone, and found that they amount to twenty-eight ; most of them, however, in order, we suppose, to increase the mystery of the thing, are set down anonymous. We were much delighted with the sight, imagining then that we had stumbled upon an ingenious critic, who had detected in the verses of the philosophical dramatist, certain recondite meanings and obscure allusions to medical subjects which had escaped the perspicacity of the Porsons and the Hermans. We fancied to ourselves that he must have succeeded in tracing the symptoms of "determination to the head," in those tremendous scenes of whirlwind passion which the poet delights in depicting. We conjectured that most probably Dr. Hull will make it appear that, when Orestes is represented as mistaking his sister for one of the furies, the poet was only describing the effects of "determination to the head ;" and that when the mad Bacchanalian women of Thebes tore Pentheus limb from limb, they were all labouring under a similar affection. It turned out, however, upon looking more narrowly into the work, that Euripides was admitted to be quite innocent of making any allusion to "determinations ;" and that it was very much in an out-of-the-way manner that his verses had been honoured with a place in the work before us. Thus, in relating the case of a poor gentleman who had hurt his health from sorrow for the death of his wife, two quotations from the *Alcestis* of Euripides, are introduced to the effect, "that it was little consolation to him that many others had been afflicted with the loss of a wife, and that his sorrow was still fresh." A quotation from the *Ion* is dragged in to adorn an anecdote of Lord Nelson, who, it appears, when he first saw the combined fleets off Trafalgar, felt as keen an appetite for his dinner as he did for fighting! (p. 19.) The principle of teetotalism is clenched with a line from Pindar, (p. 41;) and the fall of "a commercial traveller," armed with a certain bed-room utensil little calculated, one would have thought, to flourish in heroic verse, is celebrated in one of the thundering lines of Homer! (p. 23.) Quotations from Virgil, Ovid, and Martial, also occur in the work, but more sparingly, the learned author probably considering that Latin as too common for learned use.

With all its faults and peculiarities, however, we must say that the small volume before us is smartly written, and is very amusing. We think also we can perceive, through all his pedantic display of learning, that the author is a very good-natured and pleasant man; and though we can have but little sympathy with his anti-reform horrors, and do not quite understand some of his best hits (probably local), we shall not be sorry to meet with him again.

---

